# HIV-related cognitive disorders in children in Kinshasa (Democratic Republic of Congo)

**DOI:** 10.1186/s12889-024-19398-6

**Published:** 2024-07-29

**Authors:** Esther Maboso Ekolo, Aimée Masaya Mupuala, Joseph Mabiala Bodi, Adolphine Nkuadiolandu, Pierre Manyanga Tshibasu

**Affiliations:** 1 Department of Paediatrics, Faculty of Medicine, University of Kinshasa, Kinshasa, Democratic Republic of Congo; 2 Paediatric Neurology Unit, CUK, University of Kinshasa, Kinshasa, Democratic Republic of Congo; 3 Department of Gastroenterology, Nutritional Rehabilitation and Neurology, CUK, University of Kinshasa, Avenue Mangamanga 9A, Joli parc, commune de Ngaliema-Kinshasa, Kinshasa, Democratic Republic of Congo; 4 Paediatric Emergency Department, CUK, University of Kinshasa, Kinshasa, Democratic Republic of Congo; 5 Paediatrician Department of Gastroenterology, Nutritional Rehabilitation and Paediatric Neurology, CUK, University of Kinshasa, Avenue Mangamanga 9A, Joli parc, commune de Ngaliema-Kinshasa, Kinshasa, Democratic Republic of Congo

**Keywords:** HIV/AIDS (human immunodeficiency virus/acquired immune deficiency syndrome), HIV-associated neurocognitive disorder (HAND), Differential ability scale second edition (DAS-II)

## Abstract

**Objectives:**

HIV-infected individuals are at increased risk of neurocognitive disorders compared with the general population. Studies suggest that, despite the combination of antiretroviral drugs, HIV infection causes immune activation leading to significant neural damage; however, there is little data on HIV-infected young people in our country.

**Methodology:**

This is a comparative cross-sectional study conducted between November 2020 and March 2021 on two hundred and sixteen children aged 6–15 years, including 106 HIV-positive children and 108 healthy children. Cognitive performance was assessed using the Differential Ability Scale Second Edition (DAS-II).

**Results:**

HIV-infected children showed lower cognitive scores than control children in the subtest group of verbal ability (82.1% vs. 43.5%); non-verbal ability (84.9% vs. 45.4%); spatial ability (79.2% vs. 21.3%) and generall conceptual ability (GCA) (88.7% vs. 43.5%). The children in the control group had significantly higher ability scores in the diagnostic tests and in school achievement, and the difference was statistically significant.

**Conclusion:**

Cognitive impairment remains a significant complication in HIV-positive children, as suggested by low cognitive scores in more than half of our participants. This is an unresolved issue with implications for survival, quality of life and daily functioning in these children. It is important that clinicians are able to identify and manage these cognitive deficits.

## Introduction

The human immunodeficiency virus (HIV) continues to represent a major public health problem worldwide. According to UNAIDS (2019), approximately 38 million people are living with HIV. More than 32 million people have died since the beginning of the epidemic. Over the same period, children under 15 (0-14) accounted for 1.7 million cases, with 160.000 new infections and 770.000, deaths. Around 95% of new HIV infections in Middle East and North Africa [1].

The Democratic Republic of Congo (DRC) is one of the countries most affected by HIV in West and Central Africa. The HIV/AIDS epidemic in the DRC is generalised, with a prevalence in the general population of 0.8% (2019). An estimated 450,000 people are living with HIV. Children aged 0–14 account for 25% [2].

Children living with HIV face major challenges in terms of cognitive development, due to the direct neurological effects of the virus [3, 4], associated co-morbidities and unfavourable socio-economic conditions.

Cognitive impairment due to disruption of the central nervous system (CNS) by HIV has been recognised as one of the consequences of HIV infection, which can manifest as global or selective delays in neurological development. This impairment remains a significant health problem for almost half of people living with HIV, despite advances in antiretroviral treatment [5–8]. These dysfunctions range from mild neurocognitive functional impairment to severe dementia and are collectively known as HIV-associated neurocognitive disorders (HAND). The neurocognitive domains affected include processing speed, attention, executive functions, language, memory, visuo-spatial and motor skills, as well as behavioural abnormalities [5, 9, 10].

These disorders can have a significant impact on children’s ability to carry out complex activities of daily living, such as learning at school, maintaining interpersonal relationships, problem-solving and decision-making.

This can lead to personal, economic and social burdens, but above all to poor compliance with treatment.

Sub-Saharan African countries account for almost a third of neurocognitive disorders worldwide. A prevalence of 40.9% of general neurocognitive disorders was found by Lena Morgan in 2014 [11].

In the DRC, VanRie, Mupuala et al. found severe cognitive delay in 60% of HIV-positive children (2009), including 29% with significant delays in motor skills, 85% in language expression and 77% in language comprehension [12].

The impact of cognitive disorders on the overall well-being, learning and quality of life of the children affected is significant. Without appropriate intervention, these difficulties can have long-term repercussions for their future. Understanding the nature and extent of these cognitive problems in this specific context can guide the development of strategies for early detection, management and appropriate interventions to improve quality of life and well-being.

## Methodology

### Selection of participants

After establishing the selection criteria, we identified paediatric HIV care centres. A total of 214 children aged between 6 and 15 years were recruited during the period November 2020 to March 2021, divided into two groups: 106 children with known HIV and 108 children in the control group. HIV-positive children were followed up as part of an HIV care and treatment programme in three hospitals in the city and province of Kinshasa (CUK, Kalembe-lembe paediatric hospital and Centre Hospitalier Mère et Enfant Monkole). We excluded:

- All children with a history of neurological pathologies and other pathologies with an impact on the CNS (asphyxia, epilepsy, etc.);
- Children co-morbid with serious pathologies (diabetes, hepatitis C, heart disease);
- Children with an acute illness or inflammatory condition at the time of cognitive assessment.
- We worked with these centres to obtain permission to contact the families of children living with HIV and inform them about the possibility of participating in the study. Interested families gave their consent to participate in the study.

### Comparison group

Controls (HIV-negative children) were recruited from schools in the neighbourhoods where paediatric care centres specialising in HIV care existed. Age, sex and socio-economic status were taken into account. We used the same inclusion and exclusion criteria as for the HIV group. We used probability sampling of care centres and schools in the same environment.

### Neuropsychological assessment

We used the DAS-II (Differential Ability Scales second edition, Colin D. Elliot, 2007), which is a battery of cognitive tests administered individually to children and adolescents aged between 2.6 and 17.11 years. The neurocognitive battery consists of direct observation of the child performing a specific task using a battery of standardised tests, and includes accessories and toys.

### Data collection

The children were contacted one or two days before enrolment and assessment.

Sociodemographic and clinical data were extracted from the records of HIV-positive participants: CV (viral load), WHO stages, treatment regimen, date of start of antiretroviral treatment.

Sex was recorded at baseline, while weight, height and age, as well as the education level of the child and parent/guardian, were recorded on the day of assessment (for both groups).

Socio-economic status was assessed by asking questions about access to running water, the number of people in the household, the type of toilet and the regularity of income.

Cognitive functions were measured using the DAS-II (Differential Ability Scales second edition, Colin D. Elliot, 2007), which is a battery of cognitive tests administered individually to children and adolescents aged between 2.6 and 17.11 years. It consists of 20 subtests, 17 of which are cognitive and 3 are diagnostic and cognitive. The DAS-II has been approved for use in several countries around the world and correlates well with the results of other measures used in Africa, particularly in the DR Congo, such as the KABC II [13].

Short breaks of drinks and snacks were offered to the children if they became tired.

An average of 5 children were assessed per day. The duration of the assessment per child varied between 45 and 60 min, depending on the child’s behaviour. To avoid fatigue, the tests were organised over several days.

Age-adjusted standardised scores were used, calculated from the individual manual and then the DAS-II administration manual. The raw score was translated into a capacity score and then into a T score to obtain the general conceptual capacity.

This neurocognitive battery was explained in the mother tongue or in the local language for children with difficulties; the cognitive assessment was carried out by us, after having received training in the validation of this battery.

We have ensured that data confidentiality is respected and that participants’ information is anonymous.

### Data analysis

The validated data were entered into Excel and analysed using SPSS version Windows 21.0. The results are presented in the form of tables and figures. Qualitative variables are described in terms of proportions and quantitative variables in terms of measures of central tendency and dispersion (mean ± standard deviation or median with extremes).

Pearson’s chi-square test or Fisher’s exact test (for small numbers in one or more subgroups) were used to compare proportions. The Student’s t-test was used to compare the means of the variables when the distributions were Gaussian. The threshold of statistical significance was set at *p* < 0.05.

GCA: General Conceptual Ability, which provides an estimate of the child’s overall cognitive ability, was used to assess three types of ability: verbal, non-verbal and spatial.

## Result

We evaluated a total of 214 children, 106 HIV-positive and 108 non-HIV-positive, living in the community. The median age of HIV-positive children was higher than that of the control group (145.4 ± 31.1 Vs 142.9 ± 31.5).

Children of less educated parents or guardians had an unsatisfactory cognitive score (Table 1).

**Table 1:** Socio-demographic characteristics of children and parent

Variables	All *n* = 214	Case *n* = 106	Control *n* = 108	*P*
Age (months)	144,1 ± 31,2	145,4 ± 31,1	142,9 ± 31,5	0,555
Gender				0,392
Male	106(49,5)	51(48,1)	55(50,9)	
Female	108(50,5)	55(51,9)	53(49,1)	
Level of education				0,102
Primary	125(58,4)	67(63,2)	58(53,7)	
Secondary	89(41,6)	39(36,8)	50(46,3)	
Parent’s status				0,105
Parent	141(65,9)	65(61,3)	76(70,4)	
Tutor	73 (34,1)	41(38,7)	32(29,6)	
Parent’s level of education				0,004
Primary	24(11,2)	16(15,1)	8(7,4)	
Secondar	113(52,8)	63(59,4)	50(46,3)	
University	77(36,0)	27(25,5)	50(46,3)	
Profession parent				0,114
Liberal	112(52,3)	63(59,4)	49(45,4)	
Civil servant	77(36,0)	33(31,1)	44(40,7)	
No profession	25(11,7)	10(9,4)	15(13,9)	
NSE				< 0,001
Low	55(25,7)	38(35,8)	17(15,7)	
Medium	102(47,7)	60(56,6)	42(38,9)	
High	57(26,6)	8(7,5)	49(45,4)	

HIV-infected children and healthy children were well matched in terms of sex and age. The healthy subjects had a moderately high NSE (NSE : socioeconomic level) compared with the sick children, and the difference was statistically significant

Lower scores were observed in HIV-positive children in all cognitive domains assessed. The majority of subjects in the control group had average cognitive abilities in all subgroups, and only a minority had high cognitive abilities. The difference was therefore statistically significant. Children in both groups had average cognitive abilities in all subgroups (Table 2).

**Table 2:** Children’s level of performance in the basic and GCA subtests. Interpretation of group scores

Variables	All *n* = 214	Case *n* = 106	Controle *n* = 108	*P*
**Spatial ability**				< 0,001
Low	107(50,0)	84(79,2)	23(21,3)	
Medium	100(46,7)	22(20,8)	78(72,2)	
High	7(3,3)	0(0,0)	7(6,5)	
**Verbal ability**				< 0,001
Low	134(62,6)	87(82,1)	47(43,5)	
Medium	80(37,4)	19(17,9)	61(56,5)	
**Non-verbal reasoning**				< 0,001
Low	139(65,0)	90(84,9)	49(45,4)	
Medium	74(34,6)	16(15,1)	58(53,7)	
High	1(0,5)	0(0,0)	1(0,9)	
**GCA**				< 0,001
Low	141(65,9)	94(88,7)	47(43,5)	
Medium	71(33,2)	12(11,3)	59(54,6)	
High	2(0,9)	0(0,0)	2(1,9)	

The proportions of subjects with a low score are higher among children with HIV for all groups of scores: verbal ability (82.1% vs. 43.5%); non-verbal ability (84.9% vs. 45.4%); spatial ability (79.2% vs. 21.3%) and general conceptual ability (GCA) 88.7% vs. 43.5%) and the difference is statistically significant

For diagnostic tests, there was a significant difference between the HIV-infected group and the control group. The proportion of children in the control group with average cognitive ability was higher than in the HIV-positive group. For digit recall (59.4% versus 72.2% *p* < 0.001), for immediate recall (51.2 + 9% versus 82.4%), for delayed recall (69.8% versus 94.4%) and finally for information processing speed (48.1% versus 88.9%) (Table 3).

**Table 3:** Children’s cognitive performance on diagnostic clusters

Variables	All *n* = 214	Case *n* = 106	Controle *n* = 108	*P*
**Recall of digits**				< 0,001
Low	40(18,7)	37(34,9)	3(2,8)	
Medium	141(65,9)	63(59,4)	78(72,2)	
High	33(15,4)	6(5,7)	27(25,0)	
**Recall of objects immediate**				< 0,001
Low	52(24,3)	49(46,2)	3(2,8)	
Medium	144(67,3)	55(51,9)	89(82,4)	
High	18(8,4)	2(1,9)	16(14,8)	
**Recall of delayed**				< 0,001
Low	33(15,4)	32(30,2)	1(0,9)	
Medium	176(82,2)	74(69,8)	102(94,4)	
High	5(2,3)	0(0,0)	5(4,6)	
**Speed of information processing**				< 0,001
Low	58(27,1)	55(51,9)	3(2,8)	
Medium	147(68,7)	51(48,1)	96(88,9)	
High	9(4,2)	0(0,0)	9(8,3)	

Children in the control group had higher cognitive scores than those with HIV. For digit recall: 78(72.2%) vs. 63(59.4%); for direct object recall: 89(82.4%) vs. 55(51.9); for delayed recall: 102(94.4%) vs. 74(69.8%); and for speed of information processing: 96(88.9%) vs. 51(48.1%). On the other hand, children with HIV had a low cognitive score, and the difference was statistically significant

## Discussion

In the present study, we examined and identified HIV-related cognitive impairment in children aged 6–15 years.

HIV-positive children had significantly poorer cognitive development than the control group in all cognitive domains assessed, although other factors may have influenced these impairments (viral load, duration of illness, duration of antiretroviral therapy,, parent’s level of education, low socio-economic status) [8, 14].

Neurocognitive assessment of the children using the DAS-II scale showed a low score on global cognitive assessment in the group of HIV-positive children compared with the control group matched for age, sex and socio-economic level.

These results are similar to those from the Netherlands reported by Sophie Cohen, Jacqueline et al. in 2014, where the cognitive performance of HIV-positive children was low compared with age- and sex-matched healthy controls [15]. These findings are also similar to those of Ravindran, rudula P et al., in India in 2014, who found that HIV-positive children scored lower on several neurocognitive measures than the control group (attention deficit, language, verbal learning, memory, visual-motor function, fine motor function, executive function) [16].

The poor neurocognitive functions of HIV-infected children may be explained by the following reasons:

Firstly, HIV infection may have a direct effect on neurodevelopment in the early years of life, which is the period of rapid brain development, or an indirect effect through opportunistic infections [10, 17].

HIV enters the CNS in the early stages of the disease, and the resulting inflammation of the CNS leads to chronic immune activation and thus contributes to the development of HIV-associated neurocognitive disorders. The brain can then serve as a sanctuary for replicating HIV, even when systemic viral suppression has been achieved. These cognitive disorders may persist even in patients treated with ARVs, and their adverse effects on survival and quality of life are significant [14, 18–20].

Secondly, in the MISC 2018 study, in the DRC, the Early Childhood Development Index score was 56.7%, revealing that the development of Congolese children is already impaired and associated with HIV infection, these children will still show severe cognitive impairments compared to children of the same age, but who are not infected with HIV [21].

## Conclusion

The present study has shown that, despite antiretroviral therapy, the cognitive performance of HIV-positive children remains poor compared with that of healthy children.

The DAS II scale is an appropriate tool for the cognitive assessment of children aged between 2 years and 11 months and 17 years and 11 months.

Cognitive impairment remains an important complication for HIV-positive school-age children, as suggested by the low cognitive scores of more than half of our participants. Early detection and appropriate management are essential to prevent the progression of these disorders. It is crucial to raise community awareness and work together to support children affected by these disorders and their families.

The results of this study are somewhat limited due to the small sample size, which did not allow us to generalise to the entire population of Kinshasa.

Long-term follow-up would have provided a better picture of the risk factors and determinants of these disorders.

This study is the first in our field to assess the cognitive outcome of HIV-positive children. It is one of the few in Africa to have assessed school-age children.

## Data Availability

All other data and information can be obtained on reasonable request from the corresponding author.

## Abbreviations

HIV/AIDS: human immunodeficiency virus/acquired immunodeficiency syndrome
ART: antiretroviral treatment
CNS: Central Nervous System
CUK: UNIVERSITY CLINICS OF KINSHASA
DAS-II: Differential Ability Scale Second Edition
DRC: Democratic Republic of Congo
GCA: General Conceptual Ability
HAND: HIV-associated neurocognitive disorder
MICS: Multiple Indicator Cluster Survey
SEL: Socio-economic level
